# How Much Is the Eco-Efficiency of Agricultural Production in West China? Evidence from the Village Level Data

**DOI:** 10.3390/ijerph17114049

**Published:** 2020-06-05

**Authors:** Hui Xiang, Ya Hui Wang, Qi Qi Huang, Qing Yuan Yang

**Affiliations:** 1 School of Geographical Sciences, Southwest University, Chongqing 400715, China; xhui_123@163.com (H.X.); wangyhui.15b@igsnrr.ac.cn (Y.H.W); langjuanyunshu@163.com (Q.Q.H.); 2 Chongqing Key Laboratory of Karst and Environment, Chongqing 400715, China; 3 State Cultivation Base of Eco-Agriculture for Southwest Mountainous Land, Southwest University, Chongqing 400715, China; 4 Chongqing Jinfo Mountain Field Scientific Observation and Research Station for Kaster Ecosystem, Ministry of Education, Chongqing 400715, China

**Keywords:** eco-efficiency, agricultural chemicals, data envelopment analysis, west China

## Abstract

This study evaluates the eco-efficiency of agriculture in Pupiao Town, in the Yunnan province of China, through micro-level research. The term "eco-efficiency" refers to the efficiency with which ecological resources are used to meet human needs. Interviews and field research were conducted to collect the data of the 23 villages from 2016 to 2018. The Data Envelopment Analysis model (DEA) was used for data analysis. The results were as follows: (1) The eco-efficiency scores of Pupiao Town had considerable spatial heterogeneity, exhibiting a general trend of higher in the middle and lower in the east and west, which suggested eco-efficiency may be correlated with topography and transportation. (2) The value of eco-efficiency for the entire town had considerable areas for improvement and showed a slow-growth trend. (3) Fertilizers, pesticides, agricultural diesel, agricultural carbon emission, and non-point source pollution had a significant impact on eco-efficiency, followed by agricultural labor and arable land. (4) Agricultural chemicals were primary determinants affecting eco-efficiency. Most of the factors had a stronger effect on the eastern and western regions. The study suggests that transportation should be improved to promote the conveyance of market information and the application of more efficient and productive farm methods. The most important way is to improve effective utilization and to reduce the amount of agricultural chemicals. In addition, it is necessary to offer technical training and help to support farmers in upgrading their farm operations.

## 1. Introduction

The agricultural sector is considered as the most important production sector in rural areas and remains the primary source of income and livelihood for most of the rural population in developing areas [1,2]. In addition to providing food, feed, fiber and biofuel to meet humanity’s basic needs [3], agricultural activities also lead to some adverse effects, such as biodiversity reduction [4,5], soil erosion [6,7], water pollution, and fish die-offs [8]. Aimed towards improving crop productivity, intensive agrochemicals have been used in croplands without considering the negative impact on the environment [3,9,10]. This problem requires considerable attention, particularly in developing and resource-poor economies [11]. One example is China, where the agricultural economy has rapidly developed in the last few decades [12,13]. However, due to the excessive usage of agricultural chemicals, the overall conditions of China’s rural environment have seriously deteriorated. In 2018, China’s fertilizers use was the highest in the world, accounting for approximately 30% of the global consumption and was 2.84 times greater than the United States. According to the 2014 National Soil Pollution Survey Bulletin, the total over standard rate of the soil pollution point in China is as high as 16.1%. As a result, sustainable development in agriculture has been a hot issue in various disciplines for many years [14,15].

How is agricultural sustainability measured? One effective way is to adopt the indicator of "eco-efficiency" [16,17,18]. The term "eco-efficiency" appeared in the 1990s [19], and was coined to mean the efficiency with which ecological resources are used to meet human needs [20]. In general, eco-efficiency is measured as the ratio of the output to input [21,22]. The increase of output for a given level of input, or the decrease of input for a given level of output leads to the improvement in eco-efficiency [23,24]. Agricultural eco-efficiency is aimed towards improving economic output and reducing adverse environmental impact with less input [25,26]. Thus, finding ways of improving agricultural eco-efficiency is crucial in reducing agricultural chemical inputs and achieving sustainable growth of the rural economy [27,28]. It would also directly affect the long-term sustainable development and the synergy of the rural economy and environment.

Research on agricultural eco-efficiency can be assessed in terms of content, scale, and methods. The main research contents refers to the various impacts of agricultural production, and the causes of low agricultural eco-efficiency. The use of agricultural chemicals (e.g., pesticides, fertilizers) can significantly improve the efficiency of farming operations [29] and economic benefits [30], which is critical for global food security. However, it also causes serious environmental [31] and health hazards [32,33], such as increased CO_2_ [34,35], CH_4_ emissions [36], and pollution from agricultural products [37,38,39]. Several factors affect eco-efficiency, such as the external environment (e.g., weather, the amount of rainfall, temperature, and plant disease) [40], the managers (e.g., gender, attitudes, skills, capital, and knowledge ) [41,42,43], land management policies [44,45,46,47,48], and new production technologies [49].The research scale, whether macroscale (national or international), mesoscale (provincial or regional), or microscale (neighborhoods or districts), is mainly based on the objectives of the study. For instance, macro-level studies conducted in Italy [50], Rwanda [51], Latvia [52], and the UK [53] found climate change is linked with agricultural eco-efficiency. At the mesoscale, several studies in different parts of the world, for instance, in Shandong, China [54], the southern region of Portugal, Spain [55], Punjab, Pakistan [2], and Southern Italy [16] confirmed that agricultural technological measures, effective utilization of agricultural resources, and agricultural education are positive and effective ways to improve eco-efficiency. However, at the microscale, we only found one relevant study [56]: research on Spanish farmers showing the effectiveness of agri-environmental programs in improving eco-efficiency. In terms of research methods, various techniques and approaches have been applied to examine eco-efficiency, which include Data Envelopment Analysis (DEA) Error! Bookmark not defined., Energy Analysis [57], Stochastic Frontier Analysis (SFA) [58,59], and Shadow Price Analysis [60]. Among these methods, the Slack Based Model (SBM) of the Data Envelopment Analysis (DEA) is able to incorporate environmental pollution into the analysis [61], providing a more comprehensive and objective perspective. This approach has become the mainstream method for measuring agricultural eco-efficiency [62].

Most of the studies on evaluating agricultural eco-efficiency have been focused on the relationship between agricultural development and the environment and the reasons for inefficiency. Over time, the research methods have expanded and developed, providing a wide variety of choices for different applications. However, there are still significant research blind spots that need to be addressed. One is that much of the current research has been conducted mostly at the macro- or meso-levels, while micro-level analyses have been relatively limited. Micro-level studies are essential to assess the internal mechanisms of farm operations and management and towards the optimization of the human-land relationship. Also, few studies have included measuring eco-efficiency improvements based on the input-output perspective. In response to these current research blind spots, this study was conducted to evaluate agricultural eco-efficiency using micro-level analysis. Using the town of Pupiao in Yunnan, China, as study site, we applied the DEA-SBM model to assess agricultural eco-efficiency. We then characterized the spatio-temporal pattern of eco-efficiency and analyzed the causes of agricultural eco-efficiency loss. The following research questions are addressed in this study: (1) Are there significant differences in the eco-efficiency values of the different villages? (2) What are the main factors affecting agricultural eco-efficiency? (3) How can eco-efficiency be maximized? The results of this study could serve as a theoretical reference for the rational transformation of agricultural production, contribute to the effective prevention and control of agricultural pollution, and promote the sustainable growth of the rural economy.

## 2. Materials and Methods

### 2.1. Study Areas

**Figure 1:**
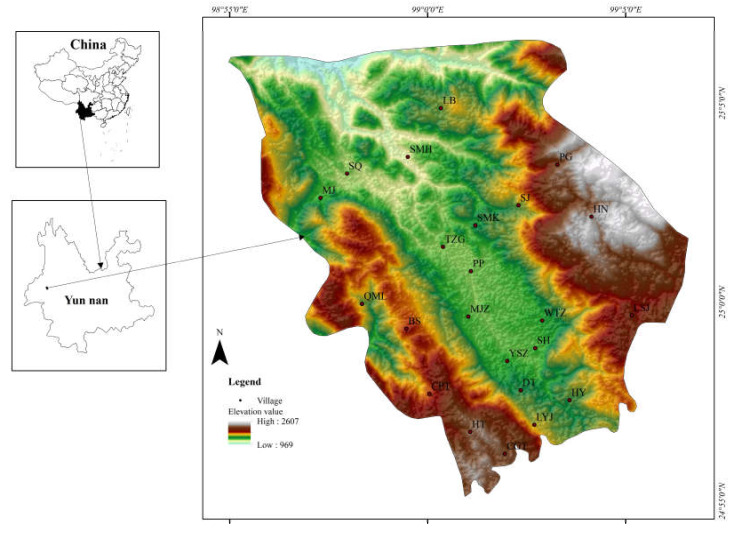
The map of the research area. The names of the villages are abbreviated: WTZ (Wangtouzhai), YSZ (Yangsanzhai), SJ (Shuijing), SH (Shuanghe), TZG (Tangzigou), PP (Pupiao), SQ (Shuangqiao), SMH (Shimuhe), LB (Luoban), SMK (Shanmenkou), HN (Huangni), PG (Pinggou), LSJ (Lengshuijing), LYJ (Liangyanjing), CGT (Cigutang), CPT (Changputang), DT (Datian), QML (Qimulin), HY (Hongyan), HT (Hetao), BS (Bingsai), MJ (Majie), and MJZ (Majiazhai).

Pupiao Town is located in Yunnan Province, west China (98°54′E–99°07′E, 24°54′N–25°06′N). Situated on the Yun-Gui Plateau (Figure 1), the town is isolated and is located 3100 km away from Beijing. Historically, it had been an important post station of the “Southern Silk Road” in ancient China. However, due mainly to its inherent remoteness and limited transportation infrastructure, the town is poorly connected with other places. The area’s topography is diverse, containing a number of mountains, hills, canyons, and basins. The general terrain can be described as high in the west and east and low in the central and north. Currently, the town has jurisdiction over 23 village committees, with a total population of more than 50,000, including 13 ethnic minorities such as the Li, Bai, and Hani. 

Pupiao has a long history of cultivation and excellent agricultural conditions. It is rich in light and heat resources, with 2076–2354 h annualsunshine hours and 4600–7800 °C active accumulated temperature ≥10 °C. Agriculture remains the most important economic sector, with more than 90% of the population working in the agricultural sector. Agriculture in Pupiao Town is dominated by planting (accounting for more than 95% of the total agricultural output value) and is supplemented by animal husbandry. The main agricultural products are vegetables, corn, rice, wheat, potato, sugarcane, and broad beans. However, Pupiao remains one of the least developed areas in China. In 2018, the per capita disposable income level of Pupiao’s rural residents was 8965 yuan ($1256), which is equivalent to 61% of the national average and 83% of the provincial mean. Since Pupiao includes mountainous areas, ethnic minority districts, agricultural zones, and poverty-stricken areas, the town is representative of many rural towns in west China. 

As with other agricultural towns, the increased urbanization in Pupiao has gradually shifted much of the population towards urban districts, creating a heightened demand for construction lands and exacerbating environmental stress. In addition, Pupiao faces a variety of problems in terms of utilization of its agricultural lands. First, the output efficiency in the agricultural lands is low. According to The Statistical Report on Agricultural Economic Information (referred to as "The Agricultural Economic Report") of 2018, the mean grain yield of the town was 8715 kg/hm^2^, which is only 70% of the national average and is also lower than the provincial mean. Second, the amount of agricultural chemicals used is relatively high. In 2018, the average fertilizers used in Pupiao was 438 kg/hm^2^, which is significantly higher than the international warning level of 255 kg/hm^2^ [63]. Thus, urgent measures are needed to optimize the use of agricultural land resources in Pupiao that would help improve eco-efficiency and support rural economic development while protecting the ecological value.

### 2.2. Data

Four datasets were used in this study. First is the general dataset of Pupiao containing information on the village committee, population, ethnic groups, and the per capita disposable income in the rural areas. The data were obtained from August 11 to 18, 2019, using interviews and field research. The second comprises data on agricultural inputs and outputs, which were obtained from The Statistical Statement of Agricultural Information of Pupiao Town (2016–2018). The third dataset is composed of administrative boundaries and DEM data and was obtained from the website of the Resource and Environment Science Data Center of the Chinese Academy of Sciences (http://www.resdc.cn/). Finally, the fourth dataset includes other pertinent information such as per capita disposable income, grain yield, and fertilizer use, which was acquired from the Chinese Bureaus of Statistics. Data related to non-point source pollution include the emissions coefficient, the loss rates of total nitrogen and total phosphorus, and ineffective utilization rates of pesticides. These values were based on The First National Pollution Source Survey—Agricultural Pollution Source Coefficient Manual (http://cpsc.mep.gov.cn/gwgg/).

### 2.3. Research Methods

#### 2.3.1. Evaluation Index System

Agriculture can refer to general agriculture (i.e., planting, animal husbandry, forestry, fishery, and others) and special agriculture (i.e., crop production). This study uses the second definition. 

Agricultural eco-efficiency is the ecological-economic performance of farms [59]. The determination of agricultural eco-efficiency indices should follow the principles of scientificity and operability. Considering the availability of indicators and actual agricultural production values, the eco-efficiency evaluation system used in this study can be classified into three aspects: input factors, expected output factors, and unexpected output factors. The total number of evaluation indicators is nine, as summarized in Table 1.

Agricultural carbon emissions and non-point source pollution were both considered as agricultural non-expected outputs. The total carbon emission can be estimated as:

(1) $$Z1=\overset{S}{\underset{1}{\sum }}F_{S}C_{S}$$

where Z1 is the total amount of agricultural carbon emissions, *F_S_* is the scale of carbon sources, and *C_S_* is the emissions coefficient. Since agricultural chemicals and tillage activities are the main sources for carbon emissions [64], four types of carbon sources have been identified: fertilizers, pesticides, tillage, and agricultural diesel. Based on existing literature [64,65], the emission coefficients for the carbon sources are as follows: fertilizers 0.8956 (t/t), pesticides 4.9341 (t/t), arable land 0.003126(t/hm^2^), and agricultural diesel 0.5927(t/t). 

Agricultural non-point source pollution is mainly derived from the use of fertilizers and pesticides. The loss in agricultural chemicals is closely linked with precipitation, farming techniques, and the natural characteristics of cultivated land. In this study, fertilizers (including nitrogen, phosphorus, compound fertilizer) and pesticides were selected as pollution sources. The proportion of N:P:K in the compound fertilizer was 1:1:1 (general compound fertilizers used in China), and the total nitrogen and phosphorus losses have been accounted for in the loss of fertilizers. In this study, the emission coefficient method was used in calculating the fertilizers loss, and the ineffective utilization rate was used to determine the pesticides residue. The formulas used in calculating these parameters are as follows:

(2) $$Z_{2h}=\overset{h}{\underset{1}{\sum }}J_{h}F_{h}C_{h\;}$$

(3) $$Z_{20}=F_{o}C_{o}$$

where *Z_2h_* is the loss of fertilizers, *J_h_* is the converted amount of total nitrogen (or total phosphorus), *F_h_* is the emissions coefficient, *C_h_* is the loss rate. *Z_2o_* is the pesticides residue, *Fo* is the pesticides usage, and *Co* is the ineffective utilization rate of pesticides.

#### 2.3.2. Eco-Efficiency of Agriculture

DEA is a method that uses a mathematical model to estimate the relative eco-efficiency of multiple inputs and outputs. The DEA-SBM model, which is non-angled and non-radial, considers the relaxation variable in the objective function and effectively solves the relaxation problem of multiple inputs and outputs. Its basic principle is as follows [66]: assume there are n decision-making units (DMU) of the agricultural system, each of which is composed of input variables (*X*) and output variables (expected outputs *Y* and unexpected outputs *Z*). Given *X* ∈ *R^m^*, *Y* ∈ *R^a^*, and *Z* ∈ *R^b^*, the categories of input factors, expected outputs, and unexpected outputs are m, a, and b, respectively. The matrices of vectors *X*, *Y* *,* and *Z* are as follows: *X* = [*X_1_, X_2_……X_n_*] ∈ *R^m^^×n^*, *Y* = [*Y_1_,Y_2_……Y_n_*] ∈ *R^a×n^*, and *Z* = [*Z_1_,Z_2_……Z_n_*] ∈ *R^b^* *^×n^*, where *X*, *Y*, and *Z* are greater than zero. Under the condition of constant returns to scale (CRS), the SBM model of production eco-efficiency is evaluated as follows:

(4) $$\left\{\begin{array}{c} \rho =min\frac{1-\frac{1}{m}\sum_{i=1}^{m}\frac{D_{i}^{-}}{X_{i}}}{1+\frac{1}{a+b}\left(\sum_{r=1}^{a}\frac{D_{r}^{e}}{Y_{r}^{e}}+\sum_{j=1}^{b}\frac{D_{j}^{k}}{Z_{j}^{k}}\right)}\\ \;\;\;\;s.t.\;\;x_{0}=X\lambda +D^{-},\;y_{0}=Y\lambda -D^{e},z_{0}=Z\lambda +D^{k}\\ D^{-}>0,D^{e}>0,D^{k}>0,>0,i=1,2\ldots m,\;\;r=1,2\ldots a,\;\;j=1,2\ldots b \end{array}\right.$$

where *ρ* is the eco-efficiency of the decision-making unit. *D^−^* is the redundancy of input, *D^e^* is the deficiency of expected output, *D^k^* is the redundancy of unexpected output, and *λ* is a constant term. Given *ρ* ∈ [0,1], the higher the value of *ρ*, the higher the eco-efficiency. When *ρ* ∈ [0,1) or when *D^−^ D^e^*, and *D^k^* are not all 0, the eco-efficiency of the decision-making units can be increased through improvements of inputs and outputs. When *ρ* = 1 and *D^−^*, *D^e^*, and *D^k^* are all 0, the eco-efficiency of the decision-making units has reached the ideal state (the minimum inputs, the minimum environmental pollution, and the maximum economic benefit). 

In addition to evaluating agricultural eco-efficiency, the DEA-SBM model can also be used to analyze the sources of eco-efficiency loss and provide practical insights for improvement. When eco-efficiency loss is present in the decision-making units, eco-efficiency can be increased by introducing agricultural improvements. Eco-efficiency loss is mainly calculated from three aspects: inputs in eco-efficiency (*IEx*), expected outputs in eco-efficiency (*IEy*), and unexpected outputs in eco-efficiency (*IEz*). The formulas are as follows:

(5) $$\;IE_{x}=\frac{1}{m}\sum_{i=1}^{m}\frac{D_{i}^{-}}{X_{i}}$$

(6) $$\;IE_{y}=\frac{1}{a+b}\sum_{r=1}^{a}\frac{D_{i}^{e}}{X_{r}^{e}}$$

(7) $$\;IE_{z}=\frac{1}{a+b}\sum_{j=1}^{b}\frac{D_{j}^{k}}{Z_{j}^{n}}$$

where *IEx* is the reducible proportion of inputs, *IEy* is the expandable proportion of expected outputs, and *IEz* is the reducible proportion of unexpected outputs. In this study, the average annual inefficiency of inputs, expected outputs, and unexpected outputs were calculated using the datasets from 2016 to 2018. Through econometric analysis, we quantitatively analyzed the allocation of inputs and outputs in order to formulate the necessary recommendations that would maximize the eco-efficiency of agriculture

## 3. Results

### 3.1. Spatial-Temporal Pattern of the Agricultural Eco-Efficiency in Pupiao Town

#### 3.1.1. Spatial Pattern of the Agricultural Eco-Efficiency

**Figure 2:**
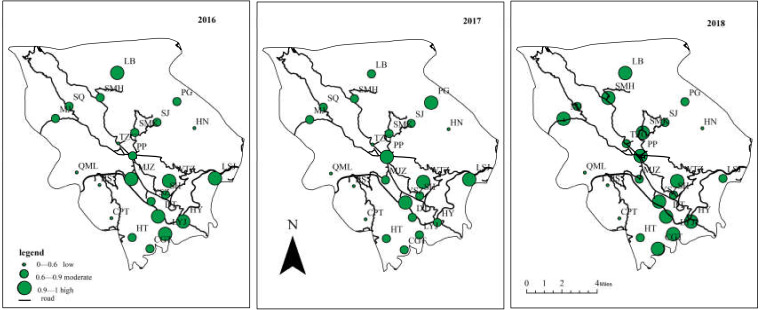
Spatial pattern of the agricultural eco-efficiency.

Using the results from agricultural eco-efficiency calculations, the charts for the spatial patterns of the town were obtained and are shown in Figure 2. Based on the value of eco-efficiency, the villages can be divided into three groups: low efficiency (lower than 0.6), moderate efficiency (0.6–0.9), and high efficiency (higher than 0.9).

The eco-efficiency scores of the villages have considerable spatial heterogeneity, exhibiting a general trend of higher in the middle and lower in the east and west. The eight villages with high scores (higher than 0.9 for at least two years) are PP, WTZ, YSZ, LB, LSY, LYJ, DT, and HY. There are five villages with low scores (lower than 0.6 for at least two years): TZG, CPT, HN, QML and BS, while the other ten villages (SH, MJZ, SMK, SJ, SQ, MJ, SMH, PG, HT, and CGT) have moderate scores. Most villages with high or moderate efficiency scores are located in the central area that has a convenient transportation system. Most villages with low-efficiency values are distributed on the east or west sides where the transportation system is underdeveloped. This reflects similarities in agricultural resource utilization and economic development among neighboring areas. In general, places with convenient transportation systems have a number of advantages, such as concentrated populations, and convenient logistics. Aside from having accessibility advantage, central Pupiao (intermontane basin) is an excellent location for sustainable development due to its excellent internal (such as technology, information) and external (such as water, soil) conditions. On the other hand, eastern and western villages (mountainous area) are more vulnerable to potential environmental problems, particularly land degradation, caused by severe soil erosion, difficulties in resource recycling, and high intensity of agricultural waste discharge.

#### 3.1.2. Temporal Pattern of the Agricultural Eco-Efficiency

**Figure 3:**
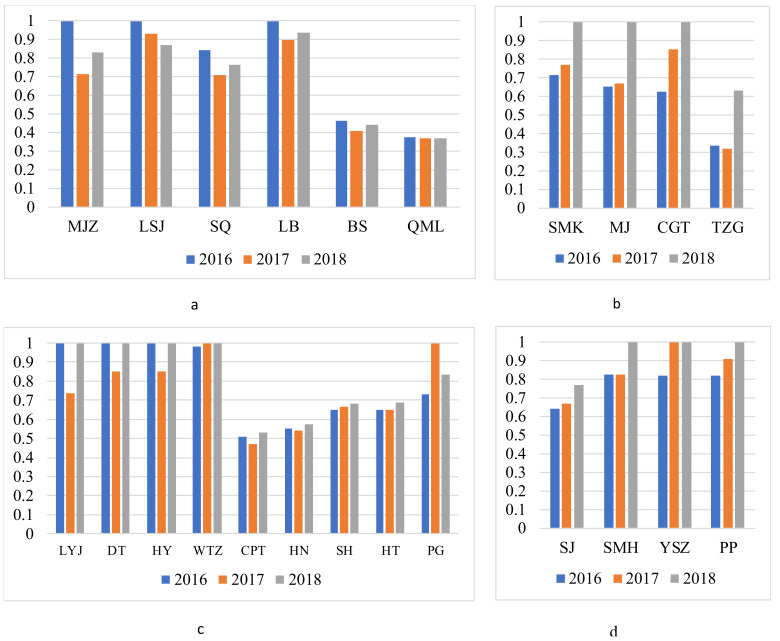
Temporal pattern of the agricultural eco-efficiency. (**a**) Villages of negative growth; (**b**) Villages of high growth; (**c**) Villages of low growth; (**d**) Villages of moderate growth.

Based on the differences in the growth rate of agricultural eco-efficiency, the villages were divided into four (Table 2): negative growth (growth rate lower than 0%), low growth (growth rate 0–5%), moderate growth (growth rate 5–10%), and high growth (growth rate higher than 10%). For comparison purposes, the village of negative growth and high growth are combined in Figure 3a,b, and the remainingtwo are in Figure 3c,d.

Over the past three years, the town, in general, did not register a high eco-efficiency score and showed a slow-growth trend. From 2016 to 2018, the average eco-efficiency levels of the town had been between 0.73 and 0.83, indicating considerable areas for improvement. The 73.92% villages showed an increasing trend (Table 2), and the growth rate rates of the villages have been unbalanced: four villages experienced high speed (Figure 3b), nine villages with low speed (Figure 3c), and four villages at medium speed (Figure 3d). This means that the overall agricultural eco-efficiency is largely improving, and considerable improvements have been achieved in agricultural production and environmental protection. In particular, the village of SMK, MJ, CGT, and TZG have shown outstanding performance, the annual increase rate for them is higher than 13% (Table 2). Unfortunately, the eco-efficiency of some villages slightly decreased during the three years (Figure 3a). For LSJ and LB, the agricultural eco-efficiency score showed distinct stability and was at the forefront of eco-efficiency, which could explain for the decrease, while the declining trend in MJZ, SQ, BS, and QML indicated substantial space to further improve resource conservation and ecological protection. Particular attention should be given to BS and QML, which have low eco-efficiency scores and descending trends. These two villages are located in the western mountainous area, where natural conditions may be unfavorable for agriculture. So, mountain areas would require more effort in order to make significant achievements in agricultural production and ecological protection.

### 3.2. Reasons for Agricultural Eco-Efficiency Loss in Pupiao Town

Using the average reducible (or expandable) proportions of the nine indicators, the sources of eco-efficiency loss in Pupiao Town were analyzed. Based on the calculated value, the indicators are divided into the following categories: low value (0–10%), moderate value (10–20%), and high value (>20%), and all of which exert a growing influence on eco-efficiency. The number and proportion of villages at different intervals for each indicator are shown in Table 3.

The agricultural eco-efficiency of the town is most affected by X3 (fertilizers), X4 (pesticides), X5 (diesel), Z1(agricultural carbon emissions), and Z2 (non-point source pollution). Due to the small number and proportion of villages with moderate value, only the high and low values need to be considered. Take X3 as an example. The number of villages with high reducible proportions is 12, and the proportion is 52.18% (Table 3). This means that about 52.18% of the villages can reduce agricultural chemicals by more than 20%, which is very serious. Similar conclusions can be drawn for X4, X5, Z1, and Z2. Part of the agricultural carbon emission comes from agricultural chemicals, and non-point source pollution is derived from the use of fertilizers and pesticides. This means agricultural chemicals are the key factors affecting the agricultural eco-efficiency of the town. In addition, X1(land) and X2 (labor) were found to have considerable impact on agricultural eco-efficiency, while Y1(grain yield) and Y2(economic yield) showed minimal effect. A significant number villages have low values for labor (13), land (15), grain yield (19), and economic crop yield (21), which suggests for many villages that these parameters have limited capacities for improvement, particularly for grain yield and economic crop yield.

This could be the result of the existing shortage of agricultural labor and cultivated land resources. Farmers tend to increase the use of agricultural chemicals in order to offset labor inputs and increase agricultural yield. Besides, Pupiao has a subtropical monsoon climate with a large amount of precipitation and is situated at a mountainous karst landform with considerable groundwater infiltration. These natural factors result in a substantial reduction of agricultural chemicals. Moreover, these findings suggest that the use and spending towards agricultural chemicals in Pupiao has become excessive so that any production advantage would be severely overshadowed by the resulting ecological and health risks.

**Figure 4:**
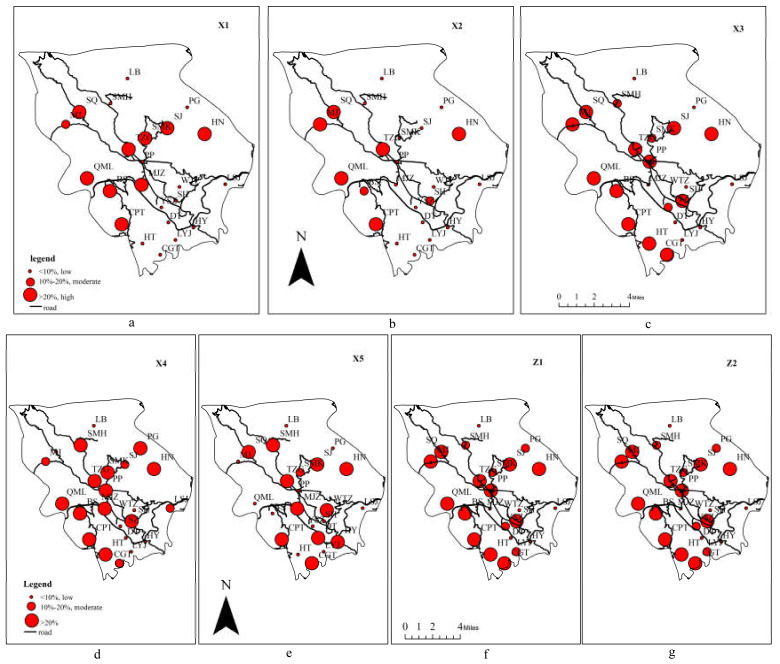
The main reasons for eco-efficiency loss. (**a**) The reducible proportion of X1; (**b**) The reducible proportion of X2; (**c**) The reducible proportion of X3; (**d**) The reducible proportion of X4; (**e**) The reducible proportion of X5; (**f**) The reducible proportion of Z1; (**g**) The reducible proportion of Z2.

Since eco-efficiency loss in the majority of the villages wasnot caused by Z1 and Z2, the two were excluded as chief sources for efficiency loss (Figure 4).

The majority of the indicators showed more substantial effect on the east and west regions. Some indicators may not be readily noticeable since the villages with high values are scattered throughout the town. For X4 (pesticides) (Figure 4d), the number of villages with high value is six for the east and west zones, and six for the central region. However, given that the east and west are mountainous areas with significantly fewer villages, the proportion of high-value villages becomes much greater. The same can be said with regard to other indicators. The greater impact on the east and west regions mainly results from the areas’ natural conditions and the underdeveloped transportation infrastructure. Due to the higher input and lower return of agricultural machinery in mountainous areas, mechanized agriculture is less practiced in the east and west regions, which results in high labor demands. With the growth in population, more cultivated lands are converted from the mountain regions, where the loss rate of agricultural chemicals is high, and environmental protection is more challenging. Moreover, the remoteness and inconvenience of travel lead to a poor exchange of agricultural products, higher market prices, and underdeveloped agricultural technology.

As for X5 (Diesel) (Figure 4e), the influence is more prominent in the central region, which differs from the other indicators. The majority of villages (70%) with high value are found in the central region, which indicates that the input of diesel in these villages can be significantly reduced. Given the large areas of cultivated land found in the mountain basin, more agricultural machinery is currently used that require high amounts of diesel.

## 4. Discussion

The results from this study suggest, similar to large-scale areas, that eco-efficiency in small-scale areas also exhibit spatial heterogeneity, which means that a one-size-fits-all policy is not applicable for micro-regions. Solving the dilemma of eco-inefficiency means improving economic output while conserving ecological service functions of the agricultural system. The main objectives are: reducing the external inputs, increasing the outputs of agricultural products, and lowering agricultural waste [67]. 

Based on the research results, the following suggestions are proposed for the mountainous areas: 

Improvement in transportation would have a positive impact on agricultural eco-efficiency. Pupiao Town’s central region, which has a more convenient transportation system, has higher agricultural eco-efficiency. This may suggest that a particular correlation exists between transportation infrastructure and agricultural eco-efficiency. Although transportation cannot improve the natural conditions, it could lead to faster market information and help to promote more efficient and productive farm methods that would improve economic performance and reduce resource waste and pollution [68]. It can also help to spread the environmental protection policy and improve the environmental protection awareness among farmers.

Improving effective utilization and reducing the amount of agricultural chemical usage are crucial strategic measures, given the problem of the excessive use of agricultural chemicals compounded by the high loss rates in mountainous areas. Inorder to reduce the use and dependency on agricultural chemicals, the following recommendations are proposed: disincentivize the use of pesticides and fertilizers in farm operations through legislation, provide subsidies that would promote the use of organic fertilizers and soil conservation measures; increase investments in agricultural science and technologies, introduce technologies and measures that would reduce agricultural carbon emissions; and optimize fertilizer utilization that would diminish agricultural pollution.

Farmers’ skills and training are also crucial aspects. It has been confirmed that many of the farmers in mountainous areas are unfamiliar with the best agricultural practices [42,43]. The government should offer technical training and help to support farmers inupgrading their farm operations. With population migration coupled with shifting age demographics, an impending shortage in agricultural labor would be critical, particularly in the mountainous areas. As a precautionary strategy, measures to make agricultural operations less reliant on heavy labor inputs should be implemented, such as promoting mechanized agriculture and modernizing other farm operations. 

In addition, the problem of excessive input in cultivated land does not need much attention. We found that it did not significantly impact eco-efficiency losses in the study area. Besides, the population in the mountainous areas has been decreasing annually due to migration, which means that the problem of rrational expansion of cultivated land will be solved gradually.

Some limitations are present in this study. Due to the unavailability of data at the village level, this study was unable to do long-term temporal analysis. At the same time, only 23 samples were included in the study. If statistics from more villages are included, more accurate results can be obtained. These areas can be explored and improved in future studies.

## 5. Conclusions

Agricultural production is still the primary source of livelihood for most rural residents in China. Due to increased agricultural pollution, various measures are needed to improve agricultural eco-efficiency. However, there is a considerable knowledge gap in the current literature regarding micro-level eco-efficiency evaluation. Using the agricultural town of Pupiao as a research area, this study used the DEA-SBM model to evaluate eco-efficiency and assessed measures to improve eco-efficiency. The main conclusions of this study are as follows:

The agricultural eco-efficiency of Pupiao Town has a higher trend in the central region and lower trend in the east and west regions. The central region generally has flat terrain and a convenient transportation infrastructure, while the eastern and western sections have rugged topography and an underdeveloped transportation system. The results suggest that eco-efficiency may be correlated with topography and transportation. The overall agricultural eco-efficiency of the town is not particularly high and shows a slow-growth trend for 2016–2018.

Fertilizers, pesticides, agricultural diesel, agricultural carbon emission, and non-point source pollution are main factors affecting Pupiao Town’s agricultural eco-efficiency. Agricultural labor and arable land also have considerable impact on eco-efficiency, while grain crop and economic crop yields have minimal effects. Agricultural chemicals (fertilizer and pesticide) are the primary determinants of eco-efficiency. A majority of the factors have a stronger impact on the eastern and western regions, while diesel has a stronger impact on the central region. 

To solve the current low eco-efficiency problem in the mountain areas, the following measures are proposed: invest in upgrading the transportation infrastructure; improve the effective utilization and reduce the dependency on agricultural chemicals; and strengthen farmers’ technical training, particularly on the use of agricultural machinery, energy-saving farming techniques, and emission-reducing farm operations.

## Figures and Tables

**Table 1:** Evaluation index system of agricultural eco-efficiency.

Target Layer	Primary Indices	Secondary Indices	Interpretation of the Indices
Eco-efficiency of agriculture	Input variables (X)	Labor input (X1) Land input (X2)	Rural employees(person) Cultivated land area (hm^2^)
		Fertilizers input (X3)	Usage of fertilizers (t)
		Pesticides input (X4)	Usage of pesticides (t)
		Diesel input(X5)	Usage of agricultural diesel (t)
	Expected output variables (Y)	Grain output(Y1)	Total grain yield (t)
		Economic crop output (Y2)	The value of economic crops (dollars)
	Unexpected output variables (Z)	Agricultural carbon emissions (Z1)	Carbon emissions from fertilizer, pesticides, etc. (t)
		Non-point source pollution (Z2)	Fertilizers loss and pesticides residue (t)

**Table 2:** The growth type of villages in Pupiao Town.

Type	Number	Proportion (%)	Amount of Change within the Three Years	Annual Growth Rate (%)
Negative growth	6	26.08	MJZ: −0.17; LSJ: −0.13 SQ: −0.08; LB: −0.07 BS: −0.02; QML: −0.01	MJZ: −5.70; LSJ: −4.27; SQ: −3.08; LB: −2.17; BS: −1.35; QML: −0.18
Low growth	9	39.13	LYJ: 0.00; DT: 0.00; HY: 0.00; WTZ: 0.02; CPT: 0.02; HN: 0.02; SH: 0.03; HT: 0.04; PG: 0.10	LYJ: 0.00; DT: 0.00; HY: 0.00; WTZ: 0.61; CPT: 1.41; HN: 1.42; SH: 1.64; HT: 1.89; PG: 4.67
Moderate growth	4	17.39	SJ: 0.12; SMH: 0.17; YSZ: 0.18; PP: 0.18	SJ: 6.39; SMH: 7.05; YSZ: 7.35; PP: 7.40
High growth	4	17.39	SMK: 0.29; MJ: 0.35 CGT: 0.38; TZG: 0.30	SMK: 13.41; MJ: 17.84; CGT: 20.09; TZG: 29.56

**Table 3:** The number and proportion of villages at different intervals of each indicator.

Type	Low Value		Moderate Value		High Value	
	Number	Proportion (%)	Number	Proportion (%)	Number	Proportion (%)
X1	13	56.52	1	4.35	9	39.13
X2	15	65.21	2	8.70	6	26.09
X3	8	34.78	3	13.04	12	52.18
X4	7	30.43	4	17.39	12	52.18
X5	12	52.18	1	4.35	10	43.47
Y1	19	82.60	2	8.70	2	8.70
Y2	21	91.30	1	4.35	1	4.35
Z1	6	26.09	5	21.74	12	52.17
Z2	6	26.09	4	17.39	13	56.52

Note: X1 is labor, X2 is land, X3 is fertilizers, X4 is pesticides, X5 is diesel, Y1 is grain output, Y2 is economic crop output, Z1 is agricultural carbon emissions, and Z2 is non-point source pollution. Same below.
